# Osteopathy in the Cranial Field as a Method to Enhance Brain Injury Recovery: A Preliminary Study

**DOI:** 10.1089/neur.2022.0039

**Published:** 2022-10-27

**Authors:** Michelle Dickerson, Susan Murphy, Natalie Hyppolite, Per Gunnar Brolinson, Pamela VandeVord

**Affiliations:** ^1^ Biomedical Engineering and Mechanics, Virginia Tech, Blacksburg, Virginia, USA.; ^2^ Edward Via College of Osteopathic Medicine, Blacksburg, Virginia, USA.; ^3^ Salem VA Medical Center, Salem, Virginia, USA.

**Keywords:** anxiety, astrocytes, blast injury, cOMM, microglia, TBI

## Abstract

The clinical burden of traumatic brain injury (TBI) continues to grow worldwide, with patients often developing chronic neurological, behavorial, and cognitive deficits. Treatment and management strategies remain a key challenge, given that they target the symptoms and not the underlying pathological response. To advance pre-clinical research and therapeutic developments, there is a need to study treatment strategies that improve brain injury recovery. Cranial osteopathic manipulative medicine (cOMM) is a non-invasive and non-pharmacological strategy that has been shown to improve quality of life for several medical conditions and injuries, and may be able to treat TBI and reduce subsequent symptoms. In this study, we aimed to evaluate the neurobiological effect of cOMM on the injury response and its potential to alleviate symptoms. We investigated the ability of cOMM to enhance fluid transport by quantifying fluorescent tracer clearance throughout the brain. Further, using an *in vivo* TBI model, male rats were exposed to a repeated blast overpressure that was followed by cOMM treatment 24 h later. Our findings indicated that cOMM treatment attenuated acute and subacute anxiety-like behaviors. Post-mortem pathological examination in the hippocampus, pre-frontal, and motor cortices indicated improvements in glial pathology in cOMM-treated animals compared to the untreated injury group. Overall, this is the first study to explore cOMM as a treatment option for brain injury, demonstrating its capability to improve TBI outcomes.

## Introduction

Traumatic brain injury (TBI) continues to be a major health concern worldwide, with more than 50 million TBI cases occurring annually.^1^ Leading causes of TBI include falls, sports, road traffic accidents, and military conflicts. Additionally, there are different mechanisms of TBI, described as impact, penetrating, and blast induced, which are heterogeneous in nature with varying injury severities.^2–4^ Mild TBI (mTBI) is the most frequently reported severity and is usually a concussion-related injury. Mild TBIs can be “invisible,” meaning there are usually no outward presenting signs of injury.^5,6^ The mechanical insult to the brain causes tissue deformation and shear stress that results in blood–brain barrier (BBB) disruption, edema, neuronal dysfunction, and inflammation.^7,8^

Further, behavioral and cognitive deficits are common sequelae post-TBI. These deficits include fear, anxiety, hypervigilance, memory loss, and depression that can persist for months, even years after injury.^9–12^ Given that patients often develop chronic, debilitating symptoms, early interventions may limit or prevent the chronification of TBI. Not only is the incidence and economic cost of TBI high, but there are also no neuroprotective/restorative drug trials that have extended past phase III clinical trials.^13,14^ Currently, treatment options are targeted toward the symptoms, not the biological injury response. Further, there is a growing interest in non-pharmacological and non-invasive treatment options to more effectively manage the clinical demand. Thus, novel treatment strategies for TBI should be explored.

An emerging strategy for mTBI recovery is cranial osteopathic manipulative medicine (cOMM). cOMM is the study of the anatomical and physiological mechanisms in the cranium and their interrelationship with the body as a whole.^15^ This includes a system of diagnostic and therapeutic manual medicine techniques, with the application to prevent and treat disease or injury. One concept of cOMM describes intrinsic rhythmic motions of the human brain, called the primary respiratory mechanism (PRM), which cause periodic fluctuations of cerebrospinal fluid (CSF) and specific relational changes among dural membranes, cranial bones, and the sacrum. cOMM techniques enhance the motion of the tissue and fluid and restore the flexibility of autonomical response by means of manipulation of the bones and sutures of the skull. cOMM enhances the flow of CSF through natural channels and helps to regulate tissue fluids of the body in general. cOMM has been successfully used as a treatment for various neurological disorders and has proven effective by cross-disciplinary studies^16–21^; however, limited data are available on the brain's response to this treatment. One therapeutic principle of cOMM is the movement of lymphatic fluid.

The recent discovery of the lymphatic system of the central nervous system (CNS; glymphatic system—a macroscopic waste clearance system in the brain) provides an opportunity to enhance brain fluid dynamics using cOMM and other lymphatic techniques. Movement of fluid through the glymphatic system could assist in regulating lymphatic tissue fluid flow of the body and thus may act as a method to enhance the clearance of waste products related to TBI within the injured brain.

Maintaining homeostasis within tissues is dependent on the clearance of excess fluid and interstitial solutes. Further, waste management in the CNS is vital for maintaining neuronal health, connectivity, and crosstalk with other cell types such as microglia and astrocytes.^22–24^ Because CNS injuries and diseases can be associated with the accumulation of cellular waste products, treatment options that enhance fluid flow and promote rapid waste clearance to improve brain health are imperative. Research has also shown that accumulated waste products within the brain parenchyma and CSF are attributable to activation of microglia and astrocytes post-injury or within the diseased brain.^25^ A study examining increased secretion of apolipoprotein E4 (APOE4) in activated astrocytes and microglia found that APOE4 expression on glial cells increased Tau protein concentration, an extracellular protein known to be involved in late-onset Alzheimer's disease (AD).^26^

Results of this study indicated that removal of astrocytic APOE4 mitigated tau-mediated neurodegeneration through decreases in tau-induced synaptic loss and microglial phagocytosis. Upregulation of proteins such as APOE4 and Tau are also prevalent in pathological conditions post-TBI, which can lead to a long-term deficit.^27^ These studies suggest that a therapeutic strategy addressing the rapid clearance of intra- and extracellular proteins could reduce astrocyte reactivity and microglia activation, thus promoting recovery of the injured brain.

Aquaporin 4 (AQP4), a water channel protein located at high density on the end-feet of astrocytes, is important in the transport of water within the perivascular spaces and clearance of metabolic waste.^28^ Abnormal expression of AQP4 related to the reactivity of astrocytes attributable to TBI could significantly impact edema, glymphatic influx, and drainage through the lymph nodes.^29–31^ Further, studies have shown that AQP4 expression plays a role in the proinflammatory response in neurological disorders, with proinflammatory states prevalent post-TBI.^32^ This could be attributable to either an increase in infiltrating peripheral immune cells, such as monocytes and leukocytes, or the activation of resident astrocytes and microglia. Monocyte chemoattractant protein 1, a chemoattractant cytokine, can recruit several leukocytes and monocytes during an immune response that then express proinflammatory cytokines, such as tumor necrosis factor alpha, interleukin (IL)-1β, and IL-8, causing further secondary damage to the brain.^33,34^ Microglia and astrocytes can also express receptors such as Toll-like receptor 4 that can recruit leukocytes within the brain, contributing to further injury.^35^

Once circulating through the brain, inflammatory cells are removed by the clearance of waste to the lymph nodes. Osteopathic physicians use the lymphatic pump technique (LPT) to improve lymphatic circulation. When used in combination with the CV4 (compression of the fourth ventricle) technique, which is designed to enhance brain fluid flow, it allows for increased mobilization of leukocytes and monocytes into lymphatic ducts, hence removing proinflammatory cytokines and aiding in injury recovery.^20,36–38^ Increased fluid mobilization may also influence AQP4 expression, which could lead to insights into mechanistic changes that result from cOMM.

cOMM may serve a crucial therapeutic role in the symptoms and progression of various neurological disorders through the physical facilitation of CNS lymphatic and CSF circulation. cOMM could aid in understanding the physiological benefits of waste clearance in improving clinical outcomes for TBI patients. However, there are no published data regarding how cOMM affects interstitial fluid flow, reduction of glial activation, or clearance of inflammatory molecules from the brain. Further, chronification of neurological and behavioral symptoms (e.g., pain, increased risk-taking behavior, depression, anxiety, self-medication, and anger) after mTBI represents a significant clinical burden leading to higher utilization of healthcare services, functional disabilities, and vocational issues.

It is essential to identify and characterize the neurobiological mechanism of cOMM so that effective clinical treatment strategies can be developed. To study the mechanisms of action of cOMM on the brain and uncover the neurological benefits after cOMM, we used the technique as a treatment in a rodent model of TBI. We hypothesized that cOMM would reduce glial pathology in the injured brain, thereby preventing and/or reducing the neuropathological sequelae and behavioral deficits related to TBI. The knowledge gained may lead to improvements in the design of non-pharmacological, non-invasive treatments and standards of care for TBI patients.

## Methods

### Animal procedures

The study described herein was carried out in accordance with experimental protocols approved by the Intuitional Animal Care and Use Committee at Virginia Tech. Before experimental procedures, 10- to 12-week-old male Sprague-Dawley rats (Envigo, Dublin, VA) were acclimated for several days (12-h light/dark cycle) with food and water provided *ad libitum*.

### Cranial osteopathic manipulative medicine treatment

**FIG. 1.**
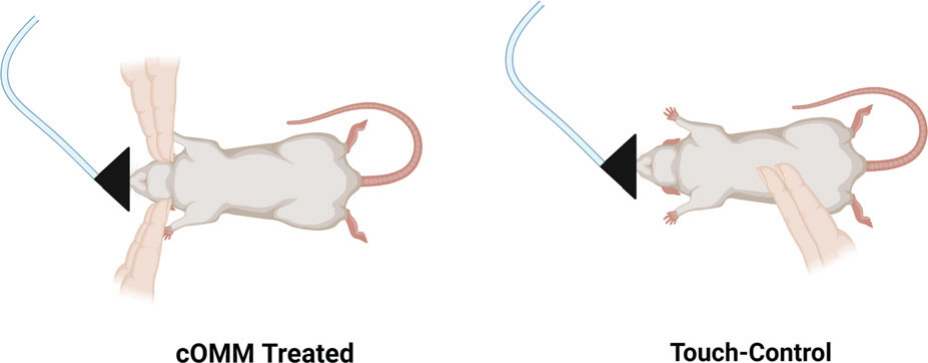
Position of the anesthetized animal while the clinician is performing the CV4 technique as a part of the cOMM treatment (left). Control animals were touched with the index and middle fingers by a non-clinician for the same amount of time as the treatment group (right). Created with BioRender.com. cOMM, cranial osteopathic manipulative medicine; CV4, compression of the fourth ventricle.

A simple CV4 technique combined with the LPT (together abbreviated as cOMM) was utilized to treat rats. The primary operator was a board-certified osteopathic physician (P.G.B., D.O.) with additional training in cOMM and experience in rodent research models. Animals were anesthetized with 5% isoflurane in an induction chamber and then transferred to a nose cone where the operator performed the treatment aimed at increasing cranial lymphatic circulation and interstitial fluid flow. The CV4 technique used a very gentle application of light pressure to the musculoskeletal structures of the head. The treatment end-point was achieved when the operator felt the tissues relax (∼5 min). The operator stabilized rats' heads using the tips of the index fingers and thumbs. Using the pads of the index fingers, gentle pressure was applied over rats' occiput, medial to the junction of the occiput and temporal bone and inferior to lambda, thereby placing tension on the dural membrane in the area of the fourth ventricle (Fig. 1). This light pressure was used to discourage the flexion phase of the primary respiratory mechanism, leading to enhanced brain interstitial fluid flow.^38^

#### Lymphatic pump technique

Immediately after CV4, the LPT was performed. To perform the LPT, the operator pressed the abdomen of the anesthetized animal with the thumb on one side and the index and middle fingers on the other side of the medial sagittal plane. The fingers were placed bilaterally, caudal to the ribs. Sufficient pressure was exerted medially and cranially to compress the lower ribs until substantial resistance was produced against the diaphragm, then the pressure was released. Compressions were administered at a rate of approximately one per second for the duration of 45 sec of treatment.

### Characterizing tracer clearance

Rats were anesthetized (5% isoflurane), and the back of the head and neck was shaved and cleaned with 70% ethanol. Using a Hamilton syringe with a 26-gauge needle, cyanine 5 (Cy5) fluorescently labeled tracers (10 μL at 0.5 mg/mL in artificial CSF, Ovalbumin, Alexa Fluor™ 647 Conjugate; ThermoFisherScientific, Waltham, MA) were injected into the cisterna magna at a rate of 2.5 μL/min to ensure tracers were infused into the subarachnoid CSF. Five minutes after tracer infusion, anesthetized rodents then underwent one session of cOMM (*n* = 8). Control animals (*n* = 6) were subjected to all the same procedures except cOMM; instead, they were touched with the index and middle fingers by a non-clinician for the same amount of time as the treatment group. The inclusion of a touch-control group was to show that cOMM alone was responsible for the effects on brain response, because in humans, in some cases, touch alone can have a placebo effect.

One hour after the treatment session, rodents were transcardially perfused with 0.9% saline and 4% paraformaldehyde (PFA). Brains were then collected and post-fixed in 4% PFA for 24 h to ensure proper fixation. After fixation, brains were rinsed in phosphate-buffered saline (PBS) and then dehydrated in 30% sucrose for 48 h; they were then embedded using optimal cutting temperature (OCT) medium. Embedded brains were then sliced into sagittal sections (60 um) using a cryostat microtome (ThermoFisherScientific). To visualize penetration of tracers into the brain parenchyma, sections were prepared for imaging by minimizing autofluorescence of the brain using the Vector TrueVIEW Autofluorescence Quenching Kit. Briefly, sections were incubated in 150 μL of reagent solution for 5 min, followed by rinsing in PBS for 5 min. Tissues were then mounted on microscope slides and cover-slipped with VECTASHIELD Vibrance Antifade Mounting Medium. Sections were imaged using the EVOS FL Auto 2 Cell Imaging System (ThermoFisherScientific) at a 4 × objective power to generate whole-slice montages. Images were analyzed by a blinded investigator using ImageJ software (NIH, Bethesda, MD).

The effect of cOMM on fluid flow and drainage of CSF was analyzed by measuring fluorescence area coverage in the brain slices of each animal, expressed as mean ± standard error of the mean (SEM) for each treatment group.

### *In vivo* traumatic brain injury model and treatment design

An established blast neurotrauma model was used to induce TBI.^39,40^ Briefly, an advanced blast simulator (ABS; 200 × 30.48 × 30.48 cm) was used to simulate free-field blast exposures, inducing TBI in the animals. The ABS consisted of three sections to create, develop, and dissipate the blast wave. The helium-driven rupture of calibrated acetate membranes created the blast wave, which traveled through the test location, reaching a passive end-wave eliminator where the blast wave was dissipated through a series of baffles. A mesh sling, designed to minimize wave hindrance, suspended and immobilized the anesthetized animals within the test section of the driver, with the nose of the animal facing the blast wave, thereby enabling exclusive focus on primary blast injuries resulting from pressure changes. Pressure measurements were collected at 250 kHz using a Dash 8HF data acquisition system (Astromed, Inc, West Warwick, RI). We used a custom MATLAB script (The MathWorks, Inc., Natick, MA) to analyze and calculate the impulse and duration of the positive and negative phases and rise time.

Animals were separated into three groups: blast + cOMM treatment (*n* = 8); blast + touch (*n* = 8); and sham + touch (*n* = 8). In preparation for blast exposure, animals were anesthetized with 5% isoflurane and placed in the mesh sling inside the ABS. Animals were exposed to three static overpressure insults (16.52 ± 2.12) separated by 1 h each (3 × 1 h). Shams underwent all the procedures except the blast insult. After either sham or blast procedures, animals were observed through the recovery stages of injury and anesthesia. Twenty-four hours after blast injury, animals received treatment with either cOMM or touch-control.

### Open field test

Anxiety-like behavior was measured using the open field test (OFT) as previously described.^41^ Briefly, the animal was placed in an arena (80 × 80 cm) in a low-light room (∼6 lux), in which the animal was allowed to explore for 5 min. The tip of the nose, center of the body, and base of the tail were tracked using EthoVision XT software (Noldus Information Technology, Leesburg, VA). The investigator was not present inside the room throughout the trial. Each trial was recorded at 30 frames per second, and proper tracking was confirmed by an investigator blind to treatments. Given that anxiety is measured as thigmotaxic behavior and/or altered activity compared to sham within the open field environment, the fraction of time spent along the walls of the arena, maximum velocity, and distance traveled were calculated and used to represent anxiety-like behavior. The OFT was administered before blast exposure to acclimate animals to the arena and repeated 2 and 7 days after injury (i.e., 1 and 6 days after cOMM treatment).

### Immunohistochemistry

Seven days after blast exposure, animals were anesthetized with 5% isoflurane and transcardially perfused with 0.9% saline followed by 4% PFA. Brains were extracted and post-fixed in 4% PFA for 24 h, rinsed in PBS, and dehydrated in a 30% sucrose solution. Fixed brains were then embedded and frozen in OCT. Coronal sections (30 μm) were prepared with a cryostat microtome. Immunohistochemistry (IHC) was performed on brain sections randomly selected that contained specific regions of interest: the medial pre-frontal cortex (PFC), the primary motor cortex (MC), and the hippocampus, to evaluate levels of AQP4 (water channels), glial fibrillary acidic protein (GFAP; astrocytes), and ionized calcium-binding adaptor molecule 1 (IBA-1; microglia).

Tissue sections were rinsed with PBS three times for 5 min each, then blocked in 2% bovine serum albumin with 0.3% Triton-X 100 for 1.5 h. Sections were then incubated in a primary antibody diluted in blocking buffer overnight at 4°C (Table 1). After incubation in the primary antibody, sections were washed in PBS containing 0.3% Triton-X 100 (PBX), then incubated with secondary antibodies for 1 h (Alexa Fluor 488 antimouse IgG [GFAP] and Alexa Flour 546 antirabbit [AQP4, IBA-1]). Sections were again washed three times in PBX for 5 min; sections were mounted on slides, air-dried, and cover-slipped with Prolong Antifade Gold reagent containing 6-diamidino-2-phenylindole (1:1000; Invitrogen, Carlsbad, CA). Sections were examined using a Zeiss fluorescence microscope (Zeiss, Jena, Germany). For imaging purposes, the hippocampus was divided further into subregions including the dentate gyrus (DG), CA1, CA2, and CA3.

**Table 1.** Primary Antibodies Used for Histological Assessments

Antibody	Catalog no.	Vendor
AQP4 (1:250)	sc3273	Santa Cruz (Dallas, TX)
GFAP (1:500)	13-300	Invitrogen (Carlsbad, CA)
IBA-1 (1:500)	CP290B	Biocare Medical (Pacheco, CA)

AQP4, aquaporin-4; GFAP, glial fibrillary acidic protein; IBA-1, ionized calcium-binding adaptor molecule 1.

For all regions, two images were taken per section and two sections were stained per animal. All images were taken at 20 × magnification. Images were processed and quantified by a blinded investigator using ImageJ software (National Institute of Health, Bethesda, MD). Background noise was subtracted using the built-in ImageJ function, and then images were thresholded to isolate only pixels with a positive signal. Percentage of signal within a given image area (area fraction), number of cells in the counting frame (count per area), average cell somas normalized to the area in the region (mean area per cell), and/or fluorescence intensity were used to determine glial activation.

### Statistical analysis

All statistical analyses were performed in GraphPad Prism software (version 9; GraphPad Software Inc., La Jolla, CA). A two-way analysis of variance (ANOVA) was used to analyze differences in time and OFT parameters for all treatment groups. A one-way ANOVA was used to compare histological data of the three treatment groups. Outliers were identified by calculating the studentized residuals, excluding data points above −2 or 2. The Brown-Forsythe and Shapiro-Wilk tests were used to verify assumptions of homoscedasticity and normality, respectively. If the data failed these assumptions, either Welch's correction *t*-test or the Kruskal-Wallis non-parametric test was performed. Data with *p* < 0.05 were considered statistically significant. Histology data were normalized to respective shams. All data are presented as the mean ± SEM.

## Results

### Increased cerecrospinal fluid transport was observed in cranial osteopathic manipulative medicine animals 1 h after treatment

**FIG. 2.**
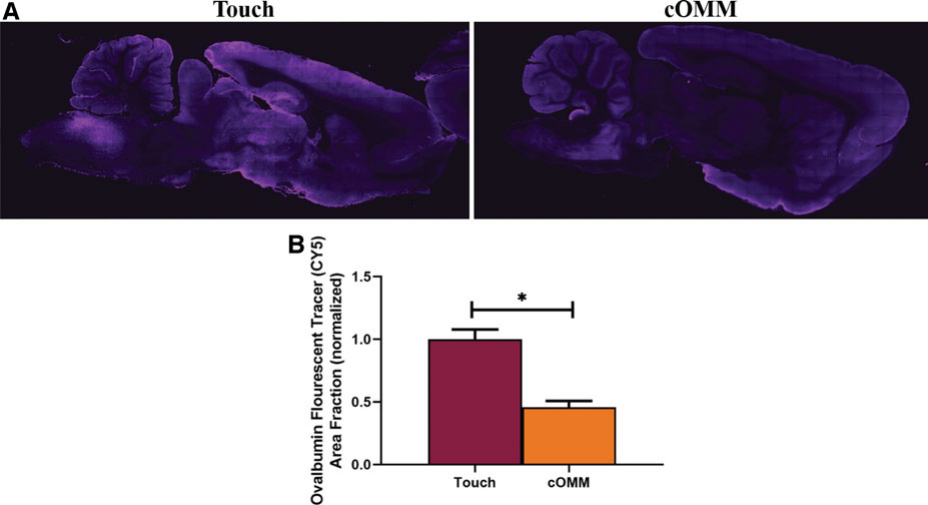
cOMM enhanced fluid flow within the brain. (**A**) Representative images show that the sham + touch group had higher levels of fluorescent tracers within the brain parenchyma compared to the cOMM group. (**B**) Quantifying tracer levels within brain tissue indicated that the area fraction of tracers in animals exposed to the sham + touch was significantly higher compared to the cOMM group. *p < 0.05. Data are represented as mean ± SEM. cOMM, cranial osteopathic manipulative medicine; SEM, standard error of the mean.

To investigate interstitial fluid flow throughout the healthy brain after cOMM, Cy5 fluorescently labeled ovalbumin molecules were delivered to the cisterna magna of anesthetized rats, followed by treatment of one session of cOMM or touch-control. The area fraction of Cy5-labeled ovalbumin tracers in brain tissue was measured as an indirect association of diffused particles to brain fluid flow in animals. The area fraction in animals exposed to the touch-control treatment was significantly higher (*p* < 0.05) compared to the cOMM group 1 h post-cOMM treatment (Fig. 2).

### Blast event and physiological outcomes

Animals were exposed to three blast events separated by 1 h each. Blast wave characteristics are described in Table 2. After each blast exposure, there were no obvious external signs of injury. One day after blast exposure, animals underwent either cOMM treatment or touch-control. There were no significant differences in weight between the three groups (mean weight) after 7 days.

**Table 2.** Blast Wave Characteristics

Group	Peak pressure (psi)	Positive duration (ms)	Positive impulse (psi^*^ms)	Rise time (ms)
3 × 1 h	16.52 ± 2.12	2.13 ± 0.22	13.34 ± 1.70	0.022 ± 0.002

Ten-week-old Sprague-Dawley rats were anesthetized and exposed to three blast insults separated by 1 h each. Sham animals underwent all procedures with the exception of the blast exposure. Data are represented as mean ± standard error of the mean.

### Anxiety-like behaviors were persistent in the untreated blast group 7 days after injury

**FIG. 3.**
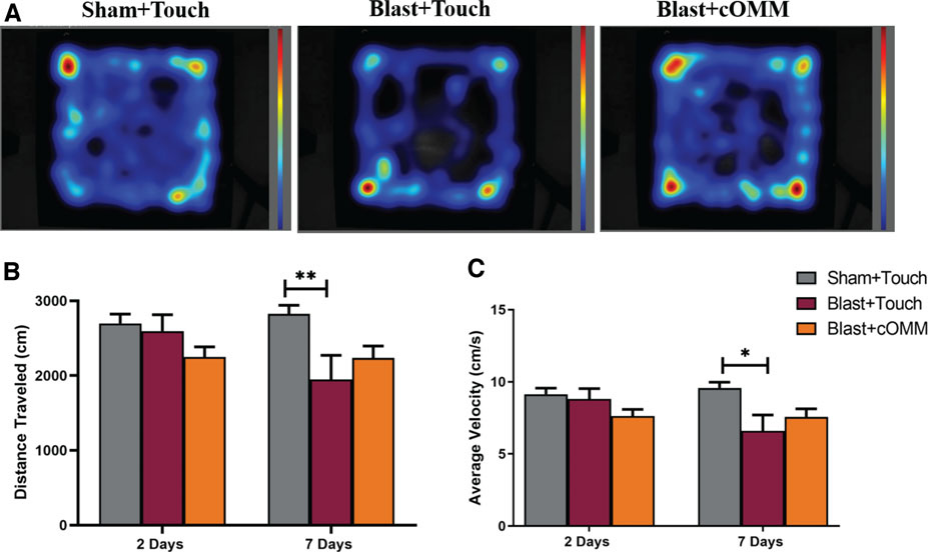
Animals were exposed to repeated blast exposure, with either “touch” or cOMM treatment being performed 24 h after bTBI. (**A**) Representative heatmap images show that the blast + touch group had a decrease in activity between the 2- and 7-day post-injury time points. (**B**) A significant interaction between time and treatment was observed for total distance traveled, with a significant decrease in total distance traveled observed in the blast + touch group in comparison to the sham + touch group 7 days after blast injury. (**C**) A significant decrease in average velocity was observed in the blast + touch group compared to the sham + touch group seven days following injury. For both total distance traveled and average velocity, a notable decrease in activity was observed in the blast + touch group, which was not observed in the sham + touch and blast + cOMM groups. *p < 0.05; **p < 0.01. Data are represented as mean ± SEM. cOMM, cranial osteopathic manipulative medicine; SEM, standard error of the mean.

Anxiety-like behaviors were observed using the OFT. Animals were exposed to a repeated blast exposure with either touch or cOMM treatment being performed 1 day post-injury. Two and 7 days after blast exposure, animals underwent the OFT (Fig. 3A). A two-way ANOVA with repeated measures indicated a significant interaction between time and treatment for total distance traveled post-injury and cOMM (*F*_(2, 21)_ = 4.527). Total distance traveled was found to be significantly decreased (*p* < 0.05) in blast + touch animals in comparison to sham + touch (Fig. 3B). A significant interaction between time and treatment was also observed for average velocity (*F*_(2, 21)_ = 4.002). A significant decrease (*p* < 0.05) in average velocity was observed in the blast + touch animals compared to the sham + touch group 7 days after injury/6 days after treatment, whereas no significant differences were found between the sham + touch and blast + cOMM groups (Fig. 3C).

### Cranial osteopathic manipulative medicine treatment restores ionized calcium-binding adaptor molecule 1 levels after blast exposure

**FIG. 4.**
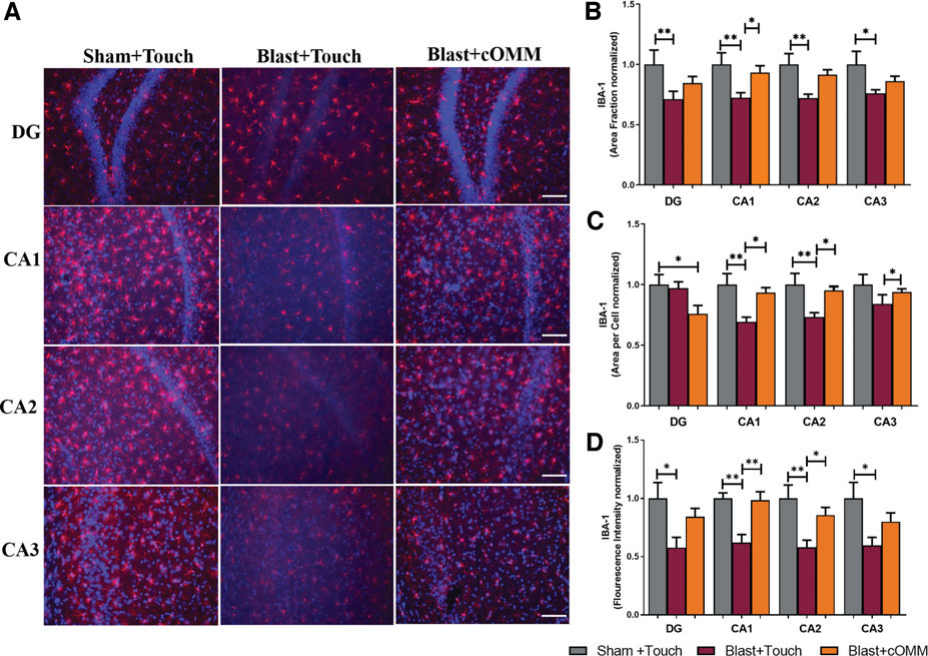
(**A**) Representative images of IBA-1 in the hippocampus region of the brain. Magnification is at 20 × and scale bar = 100 um. (**B**) Significant decreases in area fraction of IBA-1 were observed in the hippocampus of the blast + touch group compared to sham + touch. In the CA1 subregion, area fraction was significantly lower in the blast + touch group compared to both the sham + touch and blast + cOMM groups. (**C**) Mean area per cell indicated a decrease in microglia soma size in the DG subregion of the blast + cOMM group compared to the sham + touch group. In the CA1 and CA2 subregions of the hippocampus, mean area per cell was significantly lower in the blast + touch group compared to both the sham + touch and blast + cOMM groups. In the CA3 subregion, mean area per cell was significantly lower in the blast + touch group compared to the blast + cOMM group. (**D**) Fluorescent intensity of IBA-1 was significantly lower in the hippocampus of the blast + touch group in comparison to both the sham + touch and blast + cOMM groups. *p < 0.05, **p < 0.01. Data are represented as mean ± SEM. cOMM, cranial osteopathic manipulative medicine; DG, dentate gyrus; IBA-1, ionized calcium-binding adaptor molecule 1; SEM, standard error of the mean.

Measuring changes in IBA-1 was used to observe microglia activation (Fig. 4A). A one-way ANOVA indicated significant decreases in area fraction (*p* < 0.05) in blast + touch animals in comparison to both sham + touch and blast + cOMM animals in the CA1 and CA2 subregions of the hippocampus (Fig. 4B). Significant decreases in mean area per cell of microglia were observed in blast + touch animals compared to the sham + touch and blast + cOMM groups within the CA1 (*F*_(2, 21)_ = 6.985) and CA2 (*F*_(2, 21)_ = 5.658) subregions. Further, significant decreases in mean area per cell were observed in the blast + touch group in comparison to blast + cOMM in the CA3 subregion (*F*_(2, 21)_ = 4.080; Fig. 4C). A significant decrease in fluorescence intensity was observed in blast + touch animals compared to shams within the DG (*F*_(2, 21)_ = 4.356) and CA3 subregions (*F*_(2, 21)_ = 4.130). Significant decreases in fluorescence intensity were observed in the CA1 (*F*_(2, 20)_ = 10.68) and CA2 subregions (*F*_(2, 21)_ = 6.351) of the hippocampus in the blast + touch group compared to both the sham + touch and blast + cOMM groups. No significant differences were found between sham + touch and blast + cOMM for all parameters (Fig. 4D).

### Increases in ionized calcium-binding adaptor molecule 1 expression were observed in the pre-frontal cortex and motor cortex of blast animals after cranial osteopathic manipulative medicine treatment

**FIG. 5.**
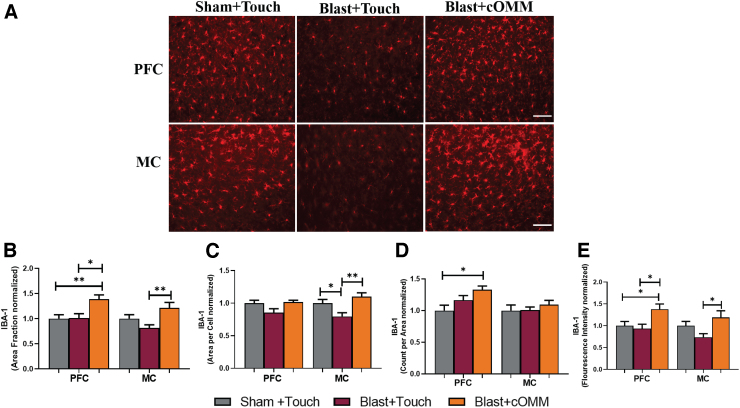
(**A**) Representative images of IBA-1 expression within the PFC and MC regions of the brain. Magnification is at 20 × and scale bar = 100 um. (**B**) Area fraction was significantly higher within the PFC of the blast + cOMM–treated animals compared to the sham + touch and blast + touch groups. Area fraction of IBA-1 was significantly higher in the MC region of the blast + cOMM group in comparison to the blast + touch group. (**C**) Mean area per cell indicated a significant decrease in microglia cell soma size in the MC of the blast + touch group compared to both the sham + touch and blast + cOMM groups. (**D**) Increased amounts of microglia were observed in the PFC of blast + cOMM animals compared to the sham + touch group. (**E**) IBA-1 expression was significantly increased in the PFC of blast + cOMM animals compared to both the blast + touch and sham + touch groups. Increases in IBA-1 expression were also observed in the MC of the blast + cOMM group compared to the blast + touch group. *p < 0.05, **p < 0.01. Data are represented as mean ± SEM. cOMM, cranial osteopathic manipulative medicine; IBA-1, ionized calcium-binding adaptor molecule 1; MC, motor cortex; PFC, pre-frontal cortex; SEM, standard error of the mean.

Significant changes in IBA-1 expression were also identified in the PFC and MC regions of the brain after cOMM treatment (Fig. 5A). A one-way ANOVA showed a significant increase (*p* < 0.05) in area fraction within the PFC (*F*_(2, 21)_ = 7.021) and MC (*F*_(2, 21)_ = 5.555) of blast + cOMM animals in comparison to the sham + touch and blast + touch groups (Fig. 5B). Area per cell of microglia was found to be significantly decreased in the MC of blast + touch animals compared to both the sham + touch and blast + cOMM groups (*F*_(2, 21)_ = 7.146; Fig. 5C). Trending decreases in microglia size were observed in the PFC of blast + touch animals compared to the blast + cOMM group (*p* = 0.06) and sham + touch group (*p* = 0.1). A significant increase in the amount of microglia (count per area) in the PFC was observed in the blast + cOMM–treated animals in comparison to the sham + touch group (*F*_(2, 21)_ = 5.075; Fig. 5D). Further, a significant increase in fluorescent intensity of the blast + cOMM treatment group was found within the PFC compared to the sham + touch and blast + touch groups (*F*_(2, 21)_ = 5.133). In the MC, fluorescent intensity was increased in the blast + cOMM group compared to the blast + touch group (*F*_(2, 21)_ = 3.932; Fig. 5E).

### Significant changes in astrocyte reactivity are observed after repeated blast exposure

**FIG. 6.**
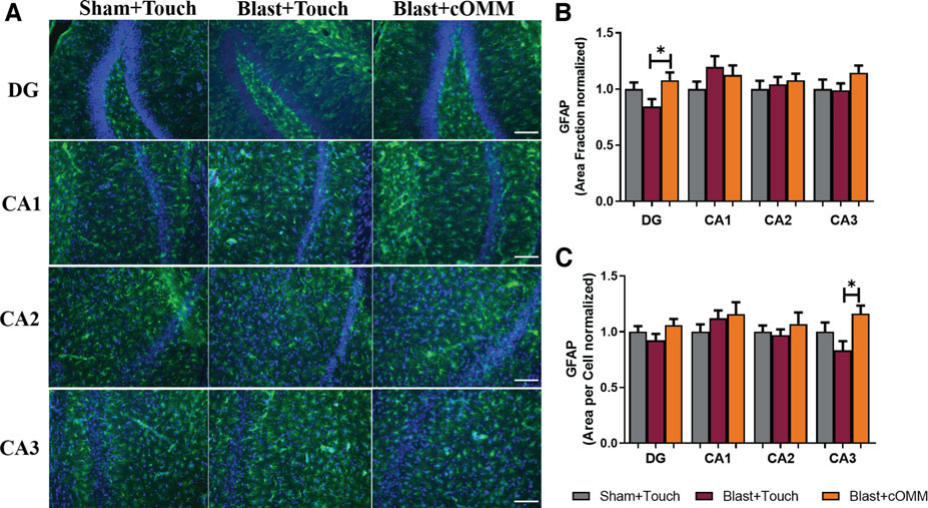
(**A**) Representative images of GFAP expression within the hippocampus. Magnification is at 20 × and scale bar = 100 um. (**B**) Area fraction was significantly decreased in the DG subregion of the hippocampus in the blast + touch group compared to the blast + cOMM group. (**C**) Decreases in cell soma size were also observed in the CA3 subregion in the blast + cOMM group compared to the blast + touch group. *p < 0.05. Data are represented as mean ± SEM. cOMM, cranial osteopathic manipulative medicine; DG, dentate gyrus; GFAP, glial fibrillary acidic protein; SEM, standard error of the mean.

Astrocyte reactivity was observed through the measurement of GFAP expression within the hippocampus (Fig. 6A). Significant decreases in area fraction of GFAP in the blast + touch animals were shown within the DG subregion of the hippocampus in comparison to blast + cOMM treatment (*F*_(2, 21)_ = 3.355; Fig. 6B). Mean area per cell indicated a significant increase in astrocyte cell size in the blast + cOMM–treated group compared to the blast + touch group in the CA3 subregion of the hippocampus (*F*_(2, 20)_ = 4.721). These differences were observed 7 days after blast exposure (Fig. 6C).

### Increases in aquaporin 4 expression were observed in the hippocampus of blast + touch animals compared to sham + touch and the blast + cranial osteopathic manipulative medicine groups

**FIG. 7.**
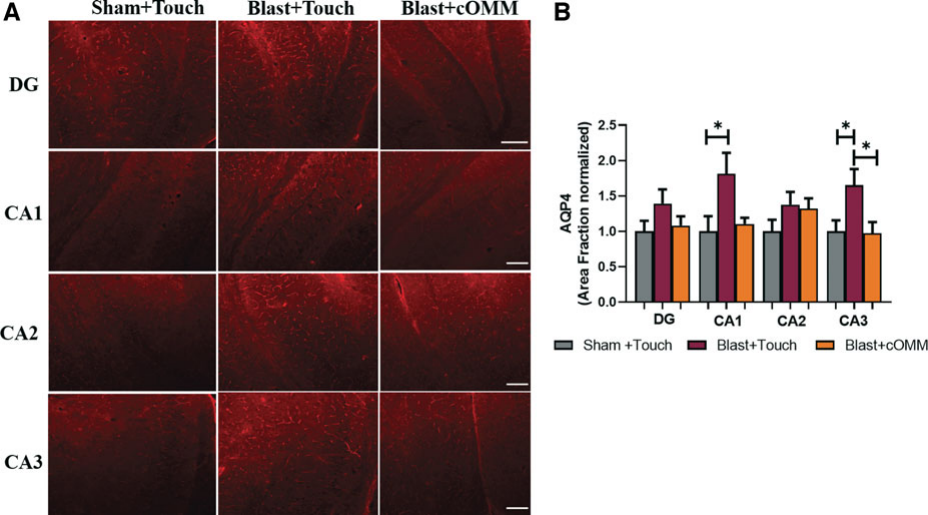
(**A**) Representative images of AQP4 in the hippocampus region of the brain. Magnification is at 10 × and scale bar = 100 um. (**B**) Within the CA1 subregion of the hippocampus, AQP4 was significantly higher in the blast + touch group compared to the sham + touch group. This increase was found to be trending (*p* < 0.1) between the blast + touch group and the blast + cOMM group. AQP4 was also found to be significantly increased in the CA3 subregion in the blast + touch group compared to both the sham + touch and blast + cOMM groups. *p < 0.05. Data are represented as mean ± SEM. AQP4, aquaporin 4; cOMM, cranial osteopathic manipulative medicine; SEM, standard error of the mean.

AQP4 is a surface protein that is highly expressed on the end-feet of astrocytes, aiding in depicting patterns of astrocyte reactivity, and even BBB integrity. AQP4 expression was measured by quantifying the area fraction in the hippocampus (Fig. 7A). Within the CA1 subregion, the area fraction of AQP4 was significantly increased in the blast + cOMM group compared to the sham + touch group (*F*_(2,19)_ = 4.117), with a trending increase in the blast + touch group compared to the blast + cOMM group (*p* = 0.08). In the CA3 subregion, a significant increase in area fraction of AQP4 was observed in the blast + touch group animals compared to both the sham + touch and blast + cOMM groups (*F*_(2, 20)_ = 4.285; Fig. 7B).

## Discussion

Treatment options for TBI are limited; therefore, it is imperative to investigate more effective approaches to improve the healthcare of TBI patients. Osteopathic physicians practice osteopathic manipulative medicine techniques to treat the symptoms of various neurological disorders.^42,43^ Despite its wide use by osteopathic physicians, the underlying mechanism for the positive effects observed after cOMM on TBI patients is not well understood. The theory is that cOMM enhances interstitial fluid flow, which is important in maintaining brain homeostatic functions. In the current study, the movement of Cy5 fluorescently labeled tracers in the brain served as an indirect association of brain fluid flow and a model for understanding interstitial fluid flow within the brain after cOMM treatment. Measurements of tracer-level diffusion in the brain parenchyma indicated that the area fraction of fluorescent tracers within the control group was significantly higher than in the cOMM treatment group. This suggests that cOMM treatment enhances fluid flow through the rapid clearance of tracers from the brain observed 1 h post-treatment.

With the combination of the CV4 and lymphatic pump techniques, manipulation of the bones and sutures of the skull as well as the lymphoid organs may have increased interstitial pressure within the brain parenchyma, pushing fluid through the brain to the lymph nodes.^44^ This may explain the decrease in tracer levels in cOMM-treated animals. Additionally, this study showed that fluid mobilization by cOMM treatment can be accomplished in a rodent model, evidence that advances the knowledge of the mechanism of action of cOMM and its treatment capabilities for TBI.

Anxiety is one comorbidity that is commonly exacerbated post-TBI.^40,45–47^ To investigate whether treatment influences subsequent behaviors post-TBI, we subjected animals to repetitive blasts separated by 1 h each; animals were then treated with either cOMM or a touch-only control 24 h post-injury. Time had a significant effect on OFT parameters. The blast + touch group showed decreased performance for total distance traveled and average velocity between the 2- and 7-day time points, whereas this notable change was not observed in the sham + touch or blast + cOMM groups. This may support the conversation around the clinical effect of osteopathic manipulative medicine on anxiety.

For example, a clinical study by Dixon and colleagues studied the effectiveness of osteopathic manipulative medicine therapy on generalized anxiety disorder (GAD).^48^ Participants with a Hamilton Anxiety Rating (HAM-A) score ≥20, indicating moderate-to-severe GAD, received five sessions of osteopathic manipulative medicine over the course of 9 weeks. After osteopathic manipulative medicine treatment, significant reductions in total HAM-A scores were observed, indicating that osteopathic manipulative medicine may be an effective alternative to conventional therapy when treating patients with GAD. This supports our claim that cOMM may have an anxiolytic effect. However, no pre-clinical studies have been conducted showing the efficacy or mechanism of action of cOMM on TBI outcomes. Our research results stress the importance of further fundamental research into cOMM to support it as an effective treatment for TBI and GAD.

Pre-clinical studies demonstrate that pathological changes in the brain post-TBI contribute to both short- and long-term sequelae such as headaches, vestibular deficits, and fear and anxiety.^49,50^ These are commonly linked to pathological changes in regions of the brain, such as the hippocampus, PFC, and MC, that play a central role in behavioral and cognitive functions.^51,52^ In the current study, IBA-1 expression and size of IBA-1^+^ microglia were significantly decreased in the cortex and hippocampus of the blast + touch group compared to the sham + touch and blast + cOMM groups.

**FIG. 8.**
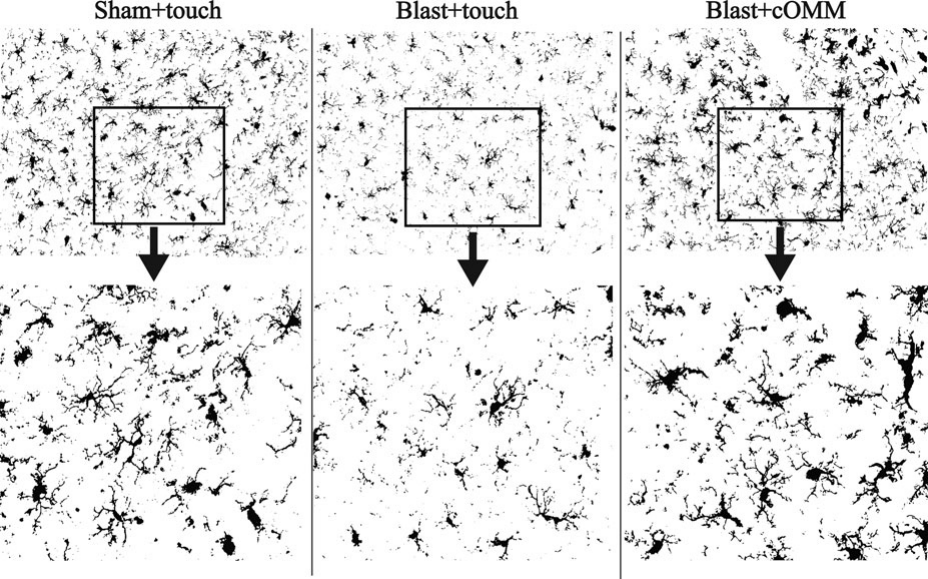
View of IBA-1^+^ microglia in the CA2 subregion of the hippocampus in the sham + touch, blast + touch, and blast + cOMM groups show various sizes of cell bodies, which may be indicative of changing morphology, a characteristic of microglia activation. Magnification is at 20 × . cOMM, cranial osteopathic manipulative medicine; IBA-1, ionized calcium-binding adaptor molecule 1.

Figure 8 is a representative image showing changes in microglia shape across injury groups within the CA2 subregion of the hippocampus, which could be contributing to significant decreases in area per cell. Further, decreases in cell size could be attributed to morphological changes that microglia undergo in response to injury, such as process retraction. For example, microglia within the blast + touch group displayed shapes that were more fragmented, with decreases in branch length, whereas microglia within the sham + touch and blast + cOMM groups were similar in morphology, with elongated processes resembling ramified morphologies. cOMM treatment could have been restoring microglia to its healthy, surveilling state, which is indicated in a mean area per cell in the blast + cOMM group that is not significanctly distinct to the sham + touch group. Decreased cell size and shape could also be representative of microglia phenotypes that decrease their expression of IBA-1, such as dystrophic or degenerating microglia, or microglia becoming more “rod-like” in response to dendritic and synaptic dysfunction.^53–56^

In contrast, levels of microglia cells and IBA-1 expression in the blast + cOMM group that resemble levels in the sham + touch group could also be attributable to the increased circulation of fluid within the brain. Given that interstitial fluid flow is enhanced, microglia may be increasing in circulation to restore the brain to pre-injury levels. A more comprehensive investigation into microglia morphology can aid in understanding the changes that microglia undergo in response to injury, contributing to a more robust analysis of distinct microglial phenotypes associated with activation, and how they adjust in response to cOMM treatment.

Further, we observed significant changes in astrocyte reactivity within the hippocampus, across the treatment groups. This finding is consistent with a pre-clinical study by Tobey and colleagues who investigated the effect of cOMM on improving the conditions related to AD, such as attenuating amyloid-β levels and reducing levels of activated astrocytes.^57^ Aged AD animals received cOMM every day for 7 days, with histological analysis revealing an increase in GFAP expression in the DG subregion, which was also observed in this pilot study. Tobey and colleagues hypothesized that with the increased circulation of fluid as a result of the cOMM treatment, astrocytes could be directly involved in these waste clearance mechanisms. Given that GFAP was also increased within the blast + cOMM group, our results support this theory.

AQP4, a water channel heavily expressed on the end-feet of astrocyte processes,^58^ was found to be decreased within the hippocampus of sham + touch and blast + cOMM animals, compared to blast + touch. In studies of pathological changes of TBI, increases in AQP4 expression correlated with BBB permeability, cerebral edema, and the facilitation of astrocyte swelling and reabsorption of edema.^59–61^ More specifically, AQP4 expression has been shown to be upregulated during the edema resolution phase at 2–14 days post-TBI, suggesting a disruption to the BBB.^59^ If cOMM enhances fluid circulation throughout the brain, we hypothesize that an increased flow of fluid throughout the brain may reduce astrocyte activation, returning AQP4 expression to pre-injury levels, given that AQP4 expression levels of the blast + cOMM and sham + touch groups were similar. In the study by Tobey and colleagues, AQP4 expression in the hippocampus was increased in AD animals treated with cOMM. Their rationale was that water channels are directly involved in the rapid waste clearance driven by cOMM, which may cause this increase in AQP4 expression.

Differences in experimental design and neurological disorders between studies contribute to varying results in brain outcomes after cOMM treatment. For example, the pathology of the aging brain and AD is very different from neurotrauma, where AQP4 would be upregulated in response to the presence of edema (excess fluid in the brain). Once edema is resolved, AQP4 levels are expected to return to baseline; therefore, pathological responses to cOMM are expected to be different. Moreover, cOMM was performed more frequently in the study by Tobey and colleagues, and did not include LPT, which may contribute to differences in the results between studies. Because of these contrasts, the various protocols of cOMM treatment should be examined to optimize the positive effects on the brain for various neurological conditions.

In the present study, a single cOMM treatment was used to improve brain injury outcomes, and it was demonstrated that cOMM increased interstitial fluid flow, which reduced the activation of glial cells. Maintaining proper fluid flow within the brain proves important in maintaining brain homeostasis, which aids in brain recovery. Further, cOMM improved anxiety-like behaviors and pathophysiology in the brain after neurotrauma. Future studies will continue to examine cOMM as a non-invasive treatment option that can improve TBI outcomes. Regional heterogeneity is also important when studying cOMM. Given that cOMM increases diffusion throughout the entire brain, studying the movement of inflammatory molecules, such as the concentration of pro- and anti-inflammatory cytokines within various regions of the brain after cOMM, is vital.

Additional studies into glial activation can provide further insight into the outcomes of cOMM on a molecular level. For example, understanding whether proteins that are expressed by microglia and astrocytes involved in engulfing waste macromolecules such as TREM2 are exacerbated or mitigated after cOMM will enhance the understanding of which areas in the brain are most sensitive to cOMM. This will relate injury recovery to behavioral outcomes that are associated with pathological changes within specific regions of the brain such as the hippocampus, PFC, and MC. Both the lymphatic system and glymphatic system are very important to brain fluid flow, and understanding the role that cOMM has on these fluid flow systems is critically important. Given that lymphatic pathways are largely influenced/regulated by water channels such as AQP4, studying lymphatic markers, especially around the fourth and lateral ventricles, is crucial to understanding the mechanism behind CNS fluid dynamics, a key component of cOMM.

To our knowledge, this is the first study to investigate cOMM as a treatment option for TBI, which is focused on understanding the mechanisms that improve acute neuropathology. This pilot study demonstrated the potential that cOMM has for the clinical management of TBI, providing a non-invasive and non-pharmacological treatment regimen as an alternative to traditional therapeutics. Subacute findings provided crucial insight on the use of cOMM as an effective treatment method for blast injury given that it decreased anxiety-like behaviors, as well as attenuated glial response after blast injury. These findings will aid in improving personalized medical interventions, which are essential for the healthcare of patients with a TBI diagnosis.

## Transparency, Rigor, and Reproducibility Statement

All projects adhere to accepted standards for rigorous study design and reporting to maximize the reproducibility and translational potential of pre-clinical research and minimize false positive/negative results.

### Randomization

Animals were labeled with an identification number and randomly assigned to one of the defined groups before the beginning of the study. All persons involved with behavior testing, cOMM treatments, and histological imaging were blinded as to which injury/treatment group animals were assigned to, minimizing subjective bias. A single board-certified osteopathic physician with experience administering cOMM to rats treated all animals.

### Power and statistical analyses

G*Power software was used to calculate group sizes. Input parameters to estimate high power for the independent groups are as follows: 1) type 1 error probability is below 5% (alpha = 0.05); 2) power is 80% (β = 0.8); and 3) effect size is 0.962 which resulted in a group size of *n* = 8 to detect both an overall significant effect of the *t*-test (Characterizing Tracer Clearance) or repeated-measures ANOVA (*In Vivo* TBI Model), and a *post hoc* difference between the blast + cOMM treatment group versus blast + touch and sham + touch controls with *p* < 0.05 after correction for multiple comparisons. For all experiments, male Sprague-Dawley rats, 10–12 weeks of age when injury was induced, were used.

#### Tracer clearance

cOMM *n* = 8, touch *n* = 6 (2 died).

#### Blast traumatic brain injury with and without cranial osteopathic manipulative medicine, histology, and behavior

Sham touch *n* = 8, Blast + touch *n* = 8, Blast + cOMM *n* = 8 for a total of 24 animals.

Post-testing analyses was performed using a two-way ANOVA to analyze the differences in time and OFT parameters for all treatment groups. A one-way ANOVA was used to compare the histological data of the three treatment groups. Outliers were identified by calculating the studentized residuals, excluding data points above −2 or 2. The Brown-Forsythe and Shapiro-Wilk tests were used to verify assumptions of homoscedasticity and normality, respectively. If the data failed these assumptions, either Welch's correction *t*-test or the Kruskal-Wallis non-parametric test was performed. Data with *p* < 0.05 were considered statistically significant. Histology data were normalized to respective shams.

## Abbreviations Used

ABS: advanced blast simulator
AD: Alzheimer's disease
ANOVA: analysis of variance
APOE4: apolipoprotein E4
AQP4: aquaporin 4
BBB: blood–brain barrier
CNS: central nervous system
cOMM: cranial osteopathic manipulative medicine
CSF: cerebrospinal fluid
CV4: compression of the fourth ventricle
Cy5: cyanine 5
GAD: generalized anxiety disorder
GFAP: glial fibrillary acidic protein
HAM-A: Hamilton Anxiety Rating
IBA-1: ionized calcium-binding adaptor molecule 1
IHC: immunohistochemistry
IL: interleukin
LPT: lymphatic pump technique
MC: motor cortex
mTBI: mild TBI
OCT: optimal cutting temperature
OFT: open field test
PBS: phosphate-buffered saline
PBX: PBS containing 0.3% Triton-X 100
PFA: paraformaldehyde
PFC: pre-frontal cortex
SEM: standard error of the mean
TBI: traumatic brain injury
